# Alarmins and c-Jun N-Terminal Kinase (JNK) Signaling in Neuroinflammation

**DOI:** 10.3390/cells9112350

**Published:** 2020-10-24

**Authors:** Nina D. Anfinogenova, Mark T. Quinn, Igor A. Schepetkin, Dmitriy N. Atochin

**Affiliations:** 1 Cardiology Research Institute, Tomsk National Research Medical Center, Russian Academy of Sciences, 634012 Tomsk, Russia; cardio.intl@gmail.com; 2 Department of Microbiology and Immunology, Montana State University, Bozeman, MT 59717, USA; mquinn@montana.edu (M.T.Q.); igor@montana.edu (I.A.S.); 3 Kizhner Research Center, Tomsk Polytechnic University, 634050 Tomsk, Russia; 4 Cardiovascular Research Center, Cardiology Division, Massachusetts General Hospital, Harvard Medical School, Charlestown, MA 02129, USA

**Keywords:** alarmin, neuroinflammation, Alzheimer’s disease, microglia, c-Jun N-terminal kinase, high-mobility group box protein 1, BAG family molecular chaperone regulator 3

## Abstract

Neuroinflammation is involved in the progression or secondary injury of multiple brain conditions, including stroke and neurodegenerative diseases. Alarmins, also known as damage-associated molecular patterns, are released in the presence of neuroinflammation and in the acute phase of ischemia. Defensins, cathelicidin, high-mobility group box protein 1, S100 proteins, heat shock proteins, nucleic acids, histones, nucleosomes, and monosodium urate microcrystals are thought to be alarmins. They are released from damaged or dying cells and activate the innate immune system by interacting with pattern recognition receptors. Being principal sterile inflammation triggering agents, alarmins are considered biomarkers and therapeutic targets. They are recognized by host cells and prime the innate immune system toward cell death and distress. In stroke, alarmins act as mediators initiating the inflammatory response after the release from the cellular components of the infarct core and penumbra. Increased c-Jun N-terminal kinase (JNK) phosphorylation may be involved in the mechanism of stress-induced release of alarmins. Putative crosstalk between the alarmin-associated pathways and JNK signaling seems to be inherently interwoven. This review outlines the role of alarmins/JNK-signaling in cerebral neurovascular inflammation and summarizes the complex response of cells to alarmins. Emerging anti-JNK and anti-alarmin drug treatment strategies are discussed.

## 1. Introduction

Neuroinflammation plays a key role in the progression or secondary injury of multiple brain conditions [1], and neuroinflammatory responses are fundamental to the pathogenesis of stroke [2], Alzheimer disease (AD), multiple sclerosis, Parkinson’s disease (PD), neurodegenerative dementias, epilepsy, psychiatric disorders, and oncologic diseases [1,3,4,5,6,7,8,9]. Sustained activation of sterile inflammatory responses occurs in cerebrovascular accidents, AD, PD, epilepsy, or traumatic brain injury, all of which involve progressive neurodegeneration [1]. The inflammatory responses associated with most chronic neurodegenerative diseases greatly depend on microglia [10]. Microglia emerge from early erythro-myeloid precursors and migrate to the brain mesenchyme prior to formation of the blood–brain barrier [11]. Genetic data implicate microglia as central players in brain health and disease [12], and microglia constitute up to a tenth of the total cell population in the brain. Being resident macrophages to the central nervous system (CNS), the microglial cells phagocytose cellular debris and foreign antigens and sense pathological events, such as inflammation [13]. Microglial cells are capable of upregulating synthesis and release of various mediators, including translocator protein (TSPO), chemokines, cyclooxygenase 1, and cannabinoid receptor 2 in the presence of inflammation. Blood-borne leukocytes, including monocytes/macrophages, neutrophils, T-lymphocytes, and B-lymphocytes extravasate into the brain through the interaction of cell surface integrins with specific endothelial adhesion molecules. Subsequently, activated cells secrete effector molecules, in particular, matrix metalloproteinases (MMP) and myeloperoxidase, which induce axonal damage and/or demyelination. Cell-to-cell interaction between the antigen-presenting cells (B-lymphocytes, microglia, and dendritic cells) occurs through CD40, amongst other molecules [14]. Microglia are crucial for neuronal wiring and functioning during health and disease. Microglial cell states are heterogeneous and context-dependent in regard to age, sex, location, and health of the surrounding neurons. External signaling factors influencing microglia, include gut microbiota and lipid metabolites, and functional clusters of microglia mutually interact with the surrounding neuronal microenvironment [12].

Danger signals released in the acute phase of ischemia trigger microglial activation, along with the infiltration of neutrophils and macrophages [15]. A significant amount of research suggests that neuroinflammation plays a causal role in AD pathogenesis, whereas understanding and control of interactions between the immune and nervous systems may eventually guide the development of therapeutics for the prevention of these diseases [16]. Indeed, neuroinflammation plays an active role in AD pathogenesis and is not just a passive response that results from the formation of senile plaques and neurofibrillar tangles [16]. This idea is supported by research demonstrating an association between microglial immune receptor expression and neuroinflammation, as well as clinical data implicating inflammation in early stages of AD development [16]. Microglia are the key innate immune cells of the CNS. While an appropriate microglial response contributes to brain tissue homeostasis and repair, an inappropriate response can lead to neural tissue damage and eventual neurodegenerative diseases [17]. Microglial cells are resident macrophage-like immune cells that are widely distributed throughout the brain and spinal cord [18,19]. Notably, microglial cells account for up to 10–15% of all cells found within various regions of the brain [18,19]. These cells represent an active immune defense system in the CNS, as they are constantly scavenging plaques, damaged or unnecessary neurons and synapses, and infectious agents [20]. Recent studies indicate that microglia also play a role in instructing and regulating the proper function of neuronal networks under healthy conditions [21]. Microglia express receptors for neurotransmitters and alarmins, also known as damage-associated molecular patterns (DAMPs) and danger signals [22,23,24]. In response to activation signals, microglia become phagocytic, antigen-presenting cells with an amoeboid morphology and produce a variety of proinflammatory and cytotoxic factors [22,25]. Since microglial cells are distributed throughout all the regions of the CNS, they have the potential to modify signaling or promote oxidative damage in neurons, either focally or globally [26]. Microglia can be activated by a number of pathological triggers, such as neuronal death or protein aggregates, resulting in their migration to sites of injury or infection where they initiate an innate immune response [16]. Thus, microglia not only orchestrate local immune responses and promote CNS healing but also have been implicated as potential effectors of neuronal injury in a variety of chronic neurodegenerative diseases, including the acquired immune deficiency syndrome dementia complex [27], AD [27,28,29], and PD [30].

Alarmins are host biomolecules that can initiate and perpetuate non-infectious and infectious inflammatory responses [31]. Alarmins are implicated in inflammatory diseases, including rheumatoid arthritis, systemic lupus erythematosus, osteoarthritis, atherosclerosis, AD, PD, and cancer. Thus, alarmins could be considered biomarkers and therapeutic targets for these diseases [32]. Alarmins are thought to be the principal sterile inflammation triggering agents. They are recognized by host cells, priming the innate immune system toward cell death and distress [1]. In stroke, alarmins act as mediators initiating the inflammatory response after their release from cellular components of the infarct core and penumbra [2]. Although alarmins are primarily considered to be soluble molecules, evidence suggests that alarmin-carrying extracellular vesicles are released from stressed or injured tissues and play a role in the induction or persistence of inflammation [33,34]. There may be a cross-talk between the alarmin-associated pathways and JNK signaling, which both are involved in control of the same processes and seem to be inherently interwoven.

The aim of this review is to outline the role of alarmins/JNK-signaling in cerebral neurovascular inflammation and to describe the complex response of the cells to alarmins. Emerging anti-JNK and anti-alarmin drug treatment strategies are also discussed.

## 2. JNK

JNKs belong to a family of mitogen-activated protein kinases (MAPK) that are triggered by various stress stimuli, including oxidative stress, heat and osmotic shock, and ischemia-reperfusion injury of the brain and heart [35,36,37,38,39]. The JNK family includes 10 isoforms encoded by JNK1 (four isoforms), JNK2 (four isoforms), and JNK3 (two isoforms) genes [40]. JNK1 and JNK2 are expressed in all cells and tissues throughout the body, whereas JNK3 is predominantly present in the heart, brain, and testicles [36]. JNKs are implicated in the pathogenesis of stroke, atherosclerosis, AD, and and PD [41,42]. JNKs are essential for the regulation of inflammation, apoptosis and necrosis signaling, and the processes involved in the neuronal injury associated with ischemia and reperfusion [41,43]. JNK-signaling plays a pivotal role in preconditioning and postconditioning of the heart and the brain [44], and studies from our group and others suggest that JNK inhibitors exert neuroprotective properties [45,46,47]. Thus, JNKs represent promising therapeutic targets for the protection of brain against ischemic stroke [44], and candidate JNK inhibitors with high therapeutic potential are currently available [44,45,48].

Downstream targets of JNK represent nearly a hundred well-defined substrates, including nuclear transcription factors (ATF2, c-Jun, Elk1, Sp1, Myc), cytoplasmic proteins regulating cytoskeletal assembly and dynamics (DCX, Tau, WDR62), vesicular transporters or JNK-interacting proteins JIP1/JIP3, transmembrane receptors such as bone morphogenetic protein receptor type 2 (BMPR2), and mitochondrial proteins (Mcl1, Bim) [43,49]. Substrates for phosphorylation-activated JNKs also include activating transcription factor 2 (ATF2), Sp1, and nuclear factors of activated T-cells (NFATc2 and NFATc3) [50,51]. Non-nuclear substrates of JNKs are involved in protein degradation, signal transduction, and regulation of cell apoptosis [37,52]. JNK is deactivated through dephosphorylation by dual specificity protein phosphatase (DUSP1/MKP-1) [53]. Folding proteins, known as JNK-interacting proteins JIP-1 and Sab, and interaction with organelles are pivotal regulators of JNK activity [44,54].

## 3. Alarmins and JNK-Signaling Cross-Talk

The recruitment and activation of antigen-presenting cells occurs early in the establishment of an immune response [55,56,57], and many microbial components and endogenous mediators participate in this process [58,59,60]. Recent studies have identified a group of structurally diverse multifunctional host proteins that are rapidly released following pathogen challenge and/or cell death and, most importantly, are able to both recruit and activate antigen-presenting cells. The potent immunostimulants include defensins, cathelicidin, eosinophil-derived neurotoxin, high-mobility group box protein 1 (HMGB1), BAG family molecular chaperone regulator 3 (BAG3), S100 proteins, heat shock proteins (Hsp), nucleic acids, histones, nucleosomes, and monosodium urate microcrystals [31,61]. They serve as early warning signals to activate both innate and adaptive immune systems. Due to the unique activities of these proteins, they are grouped under the term ‘alarmins’, in recognition of their role in mobilizing the immune system [61].

Alarmins are released from damaged or dying cells and activate the innate immune system by interacting with pattern recognition receptors [32]. These endogenous, constitutively expressed, chemotactic, and immune activating proteins/peptides are released as a result of degranulation, cell injury or death, or in response to immune induction. Alarmins are involved in a variety of processes, including regulation of antimicrobial gene expression, cellular homeostasis, wound healing, inflammation, allergy, autoimmunity, and oncogenesis [62]. The innate immune response in the brain is initiated by DAMP or pathogen-associated molecular patterns (PAMP), which are produced in response to CNS infection or injury. These molecules activate various receptors, including members of the Toll-like receptor (TLR) family, of which TLR4 is the receptor for bacterial lipopolysaccharide (LPS). Although neurons have also been reported to express TLR4, the function of TLR4 activation in neurons remains unknown [63].

Similar physiological and pathophysiological events lead to alarmin production and JNK activation. Release of alarmins seems to be one of the upstream regulatory mechanisms mediating JNK signaling. Indeed, both alarmins and JNK signaling are involved in neuroinflammation associated with numerous brain conditions [1,31,44,64] including stroke [2,44], PD [3,65], AD [1,3], epilepsy [66], traumatic brain injury [2,67], and mitochondrial dysfunction [3,66]. Mitochondrial dysfunction causes neuroinflammation through alarmin release and a series of other factors in PD, such as oxidative stress and inflammatory bodies [3]. Increased JNK phosphorylation may be involved in the mechanism of stress-induced release of endoplasmic reticulum-associated alarmins [23]. However, the interplay of alarmins/JNK signaling in neural tissue is still poorly understood.

## 4. “Find-Me” Signals

Molecularly diverse alarmins act as “find-me” signals and proinflammatory triggers [31]. Alarmins/”find-me” signals have several functions, including enhancing recognition of apoptotic cells, facilitating cleanup of apoptotic cells, and maintaining self-tolerance [31]. Apoptotic cells secrete soluble “find-me” alarmins that attract phagocytes, which are responsible for phagocytosis initiated by “eat-me” signals [68,69,70,71,72]. Although cortical glia provide trophic support to the neurons via sustained and close physical contact, “find-me” alarmins function beyond physical recruitment of phagocytes [73]. JNK is a pro-apoptotic factor, and the loss of JNK1 in hematopoietic cells protects macrophages from apoptosis and accelerates early atherosclerosis [74].

The “find-me” cue sphingosine 1-phosphate (S1P) attracts macrophages to dying cells. S1P signaling is required for efficient phagocytosis by upregulating phagocytosis receptors, including Mer receptor tyrosine kinase (MerTK) and milk fat globule epidermal growth factor VIII (MFG-E8) on macrophages [75,76]. The S1P “find-me” signal regulates both recruitment and priming of macrophages, suggesting a similarity between the effects of Spätzle (Spz5), a ligand for the Toll-1 receptor, and S1P. Being a “find-me” cue, Spz5 prepares, or primes, glia for phagocytosis in the CNS [72]. Microglial activation is involved in the pathogenesis of S1P2-mediated brain injury in mice subjected to transient middle cerebral artery occlusion. A specific antagonist of S1P2, JTE013 (1-[1,3-dimethyl-4-(2-methylethyl)-1*H*-pyrazolo[3,4-*b*]pyridin-6-yl]-4-(3,5-dichloro-4-pyridinyl)-semicarbazide), inhibits the activity of this receptor. When given orally immediately after reperfusion, JTE013 reduces the number of activated microglia and reverses their morphology from amoeboid to ramified microglia in the post-ischemic brain. It also attenuates microglial proliferation. Suppressing S1P2 activity attenuates activation of M1-relevant extracellular signal-regulated kinases 1/2 (ERK1/2) and JNK in post-ischemic brain or LPS--activated microglia. Thus, S1P signaling is considered a drug target in cerebral ischemia [77].

Injured neurons release several soluble factors, including nucleotides, cytokines, and chemokines that signal microglia to find and clear debris. Chemokine fractalkine serves as a neuronal-microglial communication factor, as shown in models of adult neurological disorders. It acts as a “find me” signal alarmin released by apoptotic neurons, and subsequently plays a critical role in modulating both clearance and inflammatory cytokine gene expression after ethanol-induced apoptosis [78]. The exact roles of fractalkine, its receptor, and microglia signaling are poorly understood in neuroinflammation. Fractalkine activates the proinflammatory pathway mediated by the transcription factor nuclear factor kappa-light-chain-enhancer of activated B cells (NF-κB) as an early response in microglial cells. Phospho-kinase assay proteome profiles indicate that fractalkine induces several kinases, including JNK [79], whose inhibition may have a neuroprotective effect [80]. JNK mediates interleukin (IL)-1β-, tumor necrosis factor (TNF)-, and interferon γ (IFN-γ)-induced fractalkine production, whereas co-incubation with JNK inhibitors suppresses fractalkine in primary human first-trimester decidual cells [81].

Efferocytosis is an apoptotic cell clearance mechanism that facilitates the removal of dangerous and damaged cells and is essential for homeostasis. Abnormal efferocytosis is associated with chronic inflammatory, autoimmune, and cardiovascular disorders, such as atherosclerosis, systemic lupus erythematous, rheumatoid arthritis, Sjogren’s syndrome, celiac disease, scleroderma, and airway inflammation [31,82,83,84,85,86,87]. In the brain, efferocytosis mediated by microglia is involved in removing excess newborn cells produced during embryonic and postnatal development in the cortex, cerebellum, and amygdala [88,89]. Microglial efferocytosis also contributes to clearing the excess cells in adult neurogenic niches in the hippocampus and subventricular zone [90,91] and dead cells during aging and neurodegenerative diseases [92]. Other cell types, such as astrocytes, neuroblasts, or neural crest cells exhibit different transcriptional and epigenetic signatures [93].

Effective and timely efferocytosis involves alarmins recognizable by macrophages and microglial cells. In the context of efferocytosis, nucleic acids, histones, nucleosomes, and monosodium urate microcrystals act as alarmins/”find-me” signals and serve as biomarkers for the prognosis and treatment of inflammatory disorders and autoimmune diseases [31]. Efferocytosis involves the interaction of receptors, bridging molecules, and apoptotic cell ligands. JNK plays a role in efferocytosis, which is essential for the pathogenesis of atherosclerosis. Loss of JNK1 in hematopoietic cells rescues macrophages from apoptosis and promotes early atherosclerosis [74], whereas scavenger receptor class B type I (SCARB1) is a critical mediator of macrophage efferocytosis via the proto-oncogene tyrosine-protein kinase (Src)/phosphoinositide 3-kinase (PI3K)/Ras-related C3 botulinum toxin substrate 1 (Rac1) pathway in atherosclerosis. Thus, agonists that resolve inflammation offer promising therapeutic potential to promote efferocytosis and prevent atherosclerotic clinical events [74]. Macrophages play a crucial role in the phagocytic clearance of dead neurons after ischemic stroke and promote the resolution of inflammation in the brain. However, the role of JNK signaling in regulation of efferocytosis during neuroinflammation is not known.

## 5. Hsp

It has been proposed that extracellular Hsp, released either through nonclassical pathways or from necrotic cells [94,95], act like alarmins, activating monocytes [96,97,98] and inducing the secretion of proinflammatory cytokines [99,100,101].

The importance of alarmin Hsp-JNK crosstalk is confirmed by the presence of a phylogenetically ancient association between these signaling systems. Indeed, caffeine-induced aversion phenotype in *Caenorhabditis elegans* is mediated by the JNK/MAPK pathway and serotonergic and dopaminergic neuroendocrine signals. RNAi depletion of Hsp-16.2, a cytosolic chaperone, reduces the aversion phenotype, suggesting that Hsp-JNK crosstalk is involved in this ancient defense mechanism [102].

The stress response is characterized in part by the upregulation of Hsp, which is compromised in neurodegenerative disorders and in some neuronal populations [103]. Since astrocytes have a greater capacity than neurons to survive metabolic stress [104] and because they are intimately associated with the regulation of neuronal function [105], it is important to understand their stress response so that we may to better appreciate the impact of stress on neuronal viability during injury or disease. It is essential to understand how stressful events alter the microenvironment that is pivotal for survival of neurons and neighboring astrocytes. Astrocytes subjected to hyperthermia upregulate the chaperone heat shock (cognate) protein 70 (HSP/c70) in addition to JNK [104]. Astrocytes release increasing amounts of Hsp/c70 into the extracellular environment following stress, an event that is abrogated when signaling through the ERK1/2 and PI3K pathways is compromised and enhanced by inhibition of the JNK pathway [104]. Extracellular Hsp70 rapidly activates JNK in macrophage RAW264.7 cells via TLR4 [106].

Hsp70, a molecular chaperone by function, has been shown to be a modulator of neurological disorders [107] and in healthy brain [108]. For example, over-expression of Hsp70 reduces paraquat-induced oxidative stress, along with JNK- and caspase-3-mediated dopaminergic neuronal cell death in a Drosophila model of neurological disorders. Likewise, over-expression of a human homologue of Hsp70, heat shock protein family A member 1 like (HSPA1L), in this model was also protective, suggesting potential relevance to humans and therapeutic applicability of Hsp70 against paraquat-induced PD-like symptoms [107]. In support of this idea, increased Hsp70 expression decreases the activated forms of JNK and p38 in the hippocampus of a rat model of fear memory consolidation [108].

Selective striatal cell death is a characteristic hallmark of the pathogenesis of Huntington’s disease [109]. Hsp have been reported to suppress the aggregate formation of mutant huntingtin and concurrent striatal cell death [110]. Geldanamycin, a benzoquinone antibiotic and Hsp90 inhibitor, exhibits protective properties against 3-nitropropionic acid-induced apoptosis and JNK activation via the induction of Hsp70 in striatal cells, suggesting that expression of Hsp70 may be a valuable therapeutic target in the treatment of Huntington’s disease. Indeed, geldanamycin significantly attenuates 3-nitropropionic acid-induced JNK phosphorylation and subsequent c-Jun phosphorylation in striatal cells [111].

Induction of Hsp inhibits both aminoglycoside- and cisplatin-induced hair cell death in whole-organ cultures of utricles from adult mice [112,113]. Celastrol, a pentacyclic triterpenoid from *Tripterygium wilfordii* induces upregulation of Hsp in utricles and provides significant protection against aminoglycoside-induced hair cell death in vitro and in vivo. Hsp32, the primary mediator of the protective effect of celastrol, inhibits pro-apoptotic JNK activation and hair cell death [112].

Hsp90 expression is significantly elevated in the retina after hydrogen sulphate (H_2_S) preconditioning and exhibits neuroprotection. ERK1/2 and JNK1-3 show specific H_2_S-dependent regulation, suggesting protective cross-talk between Hsp90 and JNK signaling [114].

## 6. HMGB1

HMGB1 is a key alarmin released upon tissue damage. HMGB1 is composed of two tandem box-like domains, Box A and Box B, each consisting of three helices [115] and is a potent immunostimulant, acting as an early warning signal to activate innate and adaptive immune systems [61]. There are at least 14 receptor systems proposed to be HMGB1 receptors [116], but only two of them, TLR4 and the receptor for advanced glycation end products (RAGE), have been verified [117,118]. Other putative receptors are most likely receptors for other molecules that bind to HMGB1, which, when expressed extracellularly, is highly inclined to bind numerous immune-activating molecules. These molecular cascades are clinically relevant regarding HMGB1-dependent inflammation. HMGB1-partner molecules include deoxyribonucleic acid (DNA), ribonucleic acid (RNA), histones, nucleosomes, LPS, stromal cell-derived factor 1 (SDF-1), IL-1α, IL-1β, and additional molecules, which are high-affinity ligands of the alleged HMGB1 receptors [119,120,121,122,123,124].

It should be noted that RAGE is also a receptor for S100 proteins and β-amyloid [125]. RAGE has been identified as an upstream regulator of JNK phosphorylation [126]. Ligand binding to RAGE increases reactive oxygen species (ROS) generation through activation of the nicotinamide adenine dinucleotide phosphate (NADPH) oxidase [127]. ROS can activate JNK in a variety of ways, and apoptosis-signal-regulated kinase 1 (ASK1) acts as a bridge in the ROS-mediated activation of the JNK pathway (see [44]).

HMGB1 is important in oxidative stress signaling, as well as in autophagy and apoptosis, whereas the mechanisms of autophagy and apoptosis in neurodegenerative diseases are associated with metabolic impairment [128]. HMGB1-JNK crosstalk is involved in autophagy, which is a tightly regulated lysosome-dependent catabolic pathway and is implicated in various pathological states in the nervous system. Autophagy is inhibited after intraperitoneal injection of anti-HMGB1 neutralizing antibodies in the rat spinal root avulsion model. HMGB1 induces autophagy and activates MAPKs, including JNK, in primary spinal neurons. Inhibition of JNK or ERK activity significantly blocks the effect of HMGB1-induced autophagy in primary spinal neurons. HMGB1-induced autophagy increases cell viability in primary spinal neurons under oxygen-glucose deprivation conditions. Therefore, HMGB1 is a critical regulator of autophagy, and HMGB1-induced autophagy plays an important role in protecting spinal neurons against injury [129]. In dopaminergic neurons in vivo, HMGB1 attenuates the decrease in tyrosine hydroxylase expression observed in the acute MPTP (methyl-4-phenyl-1,2,3,6-tetrahydropyridine) induced Parkinsonian mouse model in a JNK- and RAGE-dependent manner [130,131].

Neuronal death is replicated by exposing primary striatal neurons in culture to 3-nitropropionic acid. In rat striata intoxicated with 3-nitropropionic acid, elevations in phospho-JNK, cleaved caspase-3, and the autophagic marker LC3-II, as well as reduction in SQSTM1 (p62), are significantly reduced by the HMGB1 inhibitor glycyrrhizin. Glycyrrhizin, a triterpenoid compound from *Glycyrrhiza glabra*, also significantly inhibits 3-nitropropionic acid-induced striatal damage. 3-Nitropropionic acid, a mycotoxin, triggers the expression of HMGB1, phospho-JNK, and light chain 3-II (LC3-II) in striatal neurons, whereas phospho-JNK expression is significantly reduced by shRNA knockdown of HMGB1, an effect that is reversed by exogenously increased expression of HMGB1 [128].

Stress primes microglia by the release of alarmins, including HMGB1. HMGB1 activates the NLRP3 inflammasome, resulting in proinflammatory IL-1β production. Adult rats exposed to social defeat stress for eight days were subjected to global ischemia by four-vessel occlusion, a model for clinically relevant brain injury associated with cardiac arrest. The study showed that stress and global ischemia exerted a synergistic effect in HMGB1 release, resulting in exacerbation of NLRP3 inflammasome activation and autophagy impairment in the hippocampus of ischemic animals. Treatment with progesterone reduces HMGB1 release and NLRP3 inflammasome activation and enhances autophagy in stressed and unstressed ischemic animals. Pre-treatment with an autophagy inhibitor blocks progesterone-mediated beneficial effects in microglia. Therefore, modulation of microglial priming is one of the molecular mechanisms by which progesterone ameliorates ischemic brain injury under stressful conditions [132]. Moreover, progesterone exerts neuroprotection in AD-like rats via inhibiting β-amyloid peptide-induced activation of JNK [133].

Tyrosine hydroxylase activity reduces dopamine synthesis and is implicated in the pathogenesis of PD. HMGB1 upregulates tyrosine hydroxylase expression to maintain dopaminergic neuronal function through a mechanism dependent on JNK phosphorylation [130].

HMGB1 plays a detrimental role in hippocampal dysfunction caused by hypoxia-ischemia insult in neonatal mice [134]. Hippocampal dysfunction related to cognitive impairment and emotional disorders caused by neonatal hypoxic-ischemic brain injury in young children and adolescents has attracted increasing attention in recent years. Crosstalk between the nervous and immune systems in the context of hypoxia-ischemia injury may contribute to hippocampal dysfunction. Extracellular HMGB1 functioning as an alarmin instigates and amplifies inflammatory responses. Administration of different doses of the HMGB1-specific inhibitor glycyrrhizin reverses the hypoxia-ischemia insult-induced loss of neurons and myelin in the hippocampal region and neurobehavioral impairments. This neuroprotective effect is achieved through the inhibition of HMGB1 expression and nucleocytoplasmic translocation, a reduction in the abnormal expression of proteins associated with the downstream signaling pathway of HMGB1, a decrease in the inflammatory response, the suppression of increases in microglia/astrocytes, and the inhibition of hippocampal cell apoptosis [134]. The HMGB1 inhibitor glycyrrhizin also significantly reduces the mitochondrial inhibitor 3-nitropropionic acid-induced elevations in phospho-JNK [128].

Recent studies suggest that HMGB1 is a key alarmin with a pathogenic role in infectious diseases, such as viral or bacterial infections. HMGB1 promotes inflammatory cytokine production through RAGE, TLR2, and TLR4. HMGB1-RAGE interaction also participates in activation of ERK1/2 and JNK induced by viral infection [32,135]. HMGB1 has received attention as an alarmin by being involved in both infectious and non-infectious inflammatory conditions. Once released, HMGB1 signals through various receptors to activate immune cells. Although initial studies demonstrated HMGB1 was a late mediator of sepsis, recent findings indicate HMGB1 plays an important role in models of non-infectious inflammation, such as autoimmunity, cancer, trauma, and ischemia reperfusion injury. Moreover, unlike its proinflammatory functions, there is evidence that HMGB1 also has restorative effects, leading to tissue repair and regeneration. The complex functions of HMGB1 suggest it may be an archetypical alarmin with the potential as a target for treatment in many significant human conditions [136].

## 7. BAG3

BAG3 can act as an alarmin with different functions inside and outside the cell. c-Jun is involved in the upregulation of BAG3 [137], which is secreted by different cell types and is able to activate monocytes through binding to its membrane receptor. By interacting with heat shock protein 70 (Hsp70), BAG3 modulates the activities of this chaperone, including the delivery of client proteins to the proteasome [138]. BAG3 can also perform Hsp70-independent functions through its interactions with other proteins involved in apoptosis [139] and cytoskeletal dynamics [140,141]. Intracellularly, BAG3 sustains the levels of anti-apoptotic factors and other molecules, participates in protein quality control, drives the cytoskeleton dynamics, and exerts structural and functional roles in myocytes. In addition, the JNK pathway is associated with the protective response in kidney cancer cells against proteasome inhibition by mediating induction of BAG3 [142]. The discovery of a secreted BAG3 opened a new field of investigation on tumor development and progression, revealing a role for BAG3 in a new signaling pathway mediated by the BAG3/BAG3 receptor axis, which also includes monocytes and other stromal cells [143]. In general, BAG3 is a multifunctional protein that is involved in the cell stress response through its participation in several regulatory pathways that control cell homeostatic responses under physiological and pathological conditions [143].

Tau is a microtubule-associated protein that is found primarily in neurons. Under pathologic conditions, such as AD, tau accumulates and contributes to the disease process [144]. In rat primary neurons, activation of autophagy by inhibition of proteasome activity or treatment with trehalose results in significant decreases in tau and phospho-tau levels and induces upregulation of BAG3 [145]. Furthermore, proteasome inhibition activates JNK, which is responsible for the upregulation of BAG3 and increases tau clearance, whereas inhibition of JNK or knocking down BAG3 blocks the proteasome inhibition-induced decreases in tau. These results indicate that BAG3 plays a critical role in regulating the levels of tau in neurons, and interventions that increase BAG3 levels could provide a therapeutic approach in the treatment of AD [146].

## 8. S100 Calcium-Binding Protein B (S100B)

Microglial activation resulting from brain injury is mediated in part by alarmins, which are signaling molecules released from damaged cells [147]. The nuclear enzyme poly(ADP-ribose) polymerase-1 (PARP-1) regulates microglial activation and alarmin S100B after brain injury [147]. S100B is a protein localized predominantly to astrocytes [148] and acts either as an intracellular regulator or an extracellular signaling molecule. Exogenous S100B induces a rapid change in microglial morphology, upregulates IL-1β, TNF, and inducible nitric oxide (NO) synthase gene expression, and induces release of MMP-9 and NO in primary microglial cultures and astrocytes [149,150]. Many of these effects are attenuated in PARP-1(−/−) microglia and in wild-type microglia treated with the PARP inhibitor, veliparib (ABT-888). PARP-1 inhibition attenuates microglial activation and gene expression changes induced by S100B injected directly into brain. The anti-inflammatory effects of PARP-1 inhibitors in acutely injured brain are mediated in part through effects on S100B signaling pathways [147].

Direct correlation between the increased amount of S100B and demyelination and inflammatory processes has been demonstrated [151,152]. Pentamidine is a small molecule able to bind and inhibit S100B involved in the modulation of disease progression in a relapsing-remitting experimental autoimmune encephalomyelitis mouse model of multiple sclerosis [153]. Pentamidine can delay the acute phase of the disease and inhibit remission, resulting in amelioration of clinical score when compared with untreated relapsing-remitting experimental autoimmune encephalomyelitis mice. Moreover, pentamidine significantly reduces proinflammatory cytokines expression levels in the brains of treated versus untreated mice, in addition to reducing NO synthase activity. S100B is able to modify neuropathology, reducing immune infiltrates and partially protecting the brain from damage. Thus, pentamidine targeting S100B is considered a novel approach for multiple sclerosis treatment [153]. Indirect evidence suggests that there may be interplay between S100B and JNK signaling, as pentamidine causes activation of the JNK signaling pathway [154].

## 9. IL-33

The function of IL-33 as an alarmin has been demonstrated while studying the injury to oligodendrocytes [155], astrocytes, and microglia in the hypothalamus [156] and other glial cell-types in the CNS [155]. Neuropathic pain from injury to peripheral nerves and the CNS represents a major health care issue. Alarmin IL-33, derived from the spinal cord oligodendrocytes, mediates neuropathic pain through mechanisms involving JNK and other MAPK signaling in the experimental model of neuropathic pain in mice. Importantly, IL-33-induced hyperalgesia is markedly attenuated by inhibitors of JNK and also by inhibitors of glial cells (i.e., microglia and astrocytes) [157].

## 10. β-Amyloid

During AD pathogenesis, microglial cells bind to soluble β-amyloid oligomers and β-amyloid fibrils via cell-surface receptors resulting in an inflammatory response [26,158,159]. The β-amyloid peptide is derived from sequential enzymatic cleavage of the transmembrane region of amyloid precursor protein, resulting in a 42-amino acid fragment (also known as amyloid β_1-42_ or amyloid β_42_), which has a high tendency to form soluble oligomers and fibrils [160]. Binding of β-amyloid to a number of receptors on microglial cells results in activation of these cells to produce proinflammatory cytokines and chemokines and ROS [16]. In response to receptor engagement by β-amyloid, microglial cells phagocytose β-amyloid fibrils, which are mostly resistant to enzymatic degradation [161]. Notably, inefficient clearance of β-amyloid has been identified as a major pathogenic pathway, which may be due to increased proinflammatory cytokines and downregulation of β-amyloid phagocytic receptors [162,163].

JNK is well-known activator of the amyloidogenic pathway in AD. An increase in activation of JNK is noticeable in AD postmortem brains, suggesting a possible linkage between dysregulation of the MAPK signaling pathways and AD pathogenesis [164]. Brain tissue from humans with AD have elevated levels of Ser-phosphorylated (pSer) insulin receptor substrate 1 (IRS-1) and activated JNK. Amyloid-β peptide oligomers that accumulate in the brains of AD patients can activate the JNK pathway, induce IRS-1 phosphorylation at multiple serine residues, and inhibit physiological Tyr-phosphorylated (pTyr) IRS-1 in mature cultured hippocampal neurons [165]. In addition, JNK activation induces an intracellular β-amyloid production in neuroblastoma cells [166].

N-formyl peptide receptor (FPR)-2 has been shown to be a functional receptor for serum β-amyloid and amyloid β_42_ and thus plays a role in the neurodegenerative processes associated with of AD [167,168]. FPR2 belong to a class of G-protein-coupled receptors (GPCR). FPR2 is expressed in a wide variety of cell types, including phagocytes, hepatocytes, epithelial cells, T lymphocytes, neuroblastoma cells, microglial cells, astrocytoma cells, and microvascular endothelial cells [169]. Notably, FPRs are broadly expressed in the CNS, where FPR interactions with endogenous ligands have been implicated in the pathophysiology of several neurodegenerative diseases, including AD [170,171]. Indeed, FPR2 mediates amyloid β_42_-induced senescence in neural stem/progenitor cells in the hippocampus of APP/PS1 mice, an animal model of AD [172]. Recently, it was reported that the expression of FPR2 in primary microglial cells increased after exposure to amyloid β_42_ and that the recognition of amyloid β_42_ by FPR2 seems to initiate the signaling cascade that results in inflammation. Furthermore, Zhang et al. [173] found that FPR2 deficiency is associated with improved cognition and reduced tau phosphorylation in a mouse model of AD.

## 11. Cathelicidin (LL-37)

Cationic host defense peptides (CHDPs), also called antimicrobial peptides, function as antimicrobial and pleiotropic immunomodulatory components of innate immunity. The CHDPs comprise defensins and cathelicidins, which serve as essential innate regulators in the host tissues in mammals [174,175]. LL-37 is the only discovered member of the cathelicidin family of antimicrobial peptides in humans. LL-37 has a broad spectrum of antimicrobial activities and plays a role in various inflammatory responses [176]. LL-37 is an intrinsic immune effector and modulator present in all human tissues and is expressed in numerous cell types. Evidence suggests that LL-37 binds to amyloid β_42_ and modulates its fibril formation. Therefore, LL-37 and amyloid β_42_ naturally bind to each other, and their spatiotemporal expression balance may be essential for AD initiation and progression [177].

Little is known about an interplay between LL-37 and JNK signaling in the CNS. In other cell types, LL-37 stimulation is associated with an increase in JNK phosphorylation, and the effects of LL-37 are markedly attenuated by selective inhibitors of JNK. Therefore, MAPK signaling is involved in LL-37-mediated inhibition of inflammation [178]. In immune cells, Cdc42/Rac1-dependent bioactivity of LL-37 involves GPCR and JNK but not p38 or ERK MAPK signaling [179]. Polysaccharides from the plant *Astragalus membranaceus* are an effective immunomodulator used in the treatment of immunological diseases and can induce the expression of LL-37 in respiratory epithelial cell lines HBE16 and A549. Interestingly, *Astragalus* polysaccharides significantly elevated the phosphorylation of JNK. Furthermore, specific inhibitors of p38 MAPK, JNK, and NF-κB block *Astragalus* polysaccharide-induced LL-37 synthesis and antibacterial activity [180].

## 12. Defensins

Defensins are small cysteine-rich cationic host defense peptides displaying either direct antimicrobial activity and/or immune signaling activities. They are produced by cells of the innate immune system and epithelial cells [181]. Defensins, secreted by activated neutrophils, penetrate the blood-brain barrier, reaching into the brain and potentially contributing to neurodegeneration. Host defense peptides promote recruitment of mast cells, inducing the release of inflammatory mediators participating in blood-brain barrier disruption. This causes neuropathological changes in chronic diseases of the CNS, which further interfere with normal expression and regulatory function of defensins [175]. Impaired expression of defensins by microglia, astrocytes, choroid plexus, and pericytes may impede glymphatic fluid fluxes and prevent clearance of blood-derived neurotoxic metabolites in cases of viral infection [182,183]. Notably, expression of β-defensin 3 is increased in multiple sclerosis patients [184].

The surface layer protein of *Lactobacillus helveticus* SBT2171 stimulated β-defensin expression by activating JNK signaling via TLR2 in Caco-2 cells [185]. Flagellin-mediated β-defensin 2 induction in T84 colon carcinoma cells was significantly reduced by SP600125, an anthrapyrazolone inhibitor of JNK, but not ERK inhibitors [186]. Similar effects were found in pulmonary BEAS-2B epithelial cells, infected by *Moraxella catarrhalis*. However, phosphorylation of JNK was inhibited by β-defensin 3 in human umbilical vein endothelial cells [187]. SP600125 also significantly suppressed α-defensin-1-induced MMP-1 production in fibroblast-like synoviocytes [188]. The possible interplay between defensins and JNK signaling in neuroinflammation remains poorly understood and requires further studies.

## 13. α-Synuclein

α-Synuclein, a key neurotoxic protein involved in PD, accumulates within the endoplasmic reticulum both in animal models of α-synucleinopathy and in human PD patients. The extracellular aggregates of α-synuclein behave like alarmins, whereas the presence of autoantibodies against α-synuclein species in the cerebrospinal fluid and the serum of individuals with PD implicate the involvement of innate and adaptive immune responses [189]. α-Synuclein is suggested to have a fundamental function, both in the neuronal events occurring in PD and in the immune response during the disease. It can act directly on immune cells, including microglia, initiating a sterile response essential for neuronal health and translating in a peripheral immune response. In turn, microglia clear α-synuclein, preventing upregulation of the molecule, which is crucial to disease progression [190]. Considering that accumulation of α-synuclein is implicated in the pathogenesis of PD, enhancing its clearance might be a promising strategy in PD treatment. JNK and NF-κB signaling are responsible for the neuroinflammation during challenge with α-synuclein aggregates [191]. Thus, the JNK pathway may link the malfunction of α-synuclein with oxidative stress-triggered apoptosis, finally ascribing a common pathogenic mechanism to both the sporadic and familial forms of PD. JNK activity is pivotal in the secretory fate of autophagosomes containing α-synuclein [192], and A30P mutant α-synuclein decreases phospho-JNK levels in midbrain dopaminergic neuron [193]. In addition, the efficacy of caffeic acid on A53T α-synuclein degradation is reversed by the JNK inhibitor SP600125 [194]. Therefore, the crosstalk of α-synuclein and JNK-signaling may be a new target for future neuroprotective therapies.

## 14. Mitochondrial DNA (mtDNA)

Neuroinflammation is associated with a large array of neurological disorders where mitochondrial alarmins are a common pathway promoting disease progression [195]. Different stimuli, such as oxidative stress and impaired quality control, results in mitochondrial constituents including mtDNA displaced toward intra- or extracellular compartments. Once discarded, mtDNA may act as an alarmin and trigger innate immune inflammatory responses by binding to danger-signal receptors [195]. It is currently unknown whether there is an interplay between mtDNA- and JNK-associated signaling systems. However, oxidative stress may be the common hub where these two systems interfere with each other.

## 15. Anti-Alarmin Agents

The experimental studies and clinical trials focusing on individual alarmins involved in neurological disorders are ongoing. Alarmins/”find-me” signal molecules serve as targets for the following pharmacological agents: necrostatins, recombinant Fcnb, anti-histone, neutralizing antibodies, aminophylline, activated protein C, CD24IgG recombinant fission protein, and recombinant thrombomodulin [31].

The release of alarmins, such as myeloid-related protein 14 (MRP14) and HMGB1, maintains inflammation. Evidence suggests that paquinimod, an MRP14-inhibitor, and an anti-HMGB1 antibody can improve clinical outcome as adjunctive therapeutics in a mouse model of pneumococcal meningitis, and adjunctive inhibition of MRP14 or HMGB1 reduces mortality in mice with pneumococcal meningitis. However, this effect is lost when the two anti-DAMP agents are given simultaneously, possibly due to excessive immunosuppression. Anti-DAMP treatment alone is sufficient and superior to alternative treatment modalities [196]. Therefore, alarmin inhibition has good potential as an adjuvant treatment approach for pneumococcal meningitis, as it improves clinical outcome and can be given together with the standard adjuvant dexamethasone without loss of drug effect in experimental pneumococcal meningitis [196].

A ubiquitous nuclear protein HMGB1 promotes inflammation when released extracellularly after cellular activation, stress, damage, or death. It operates as one of the most intriguing molecules in inflammatory disorders via signaling and molecular transport mechanisms. Treatments based on antagonists specifically targeting extracellular HMGB1 have generated promising results in a wide array of experimental models of infectious and sterile inflammation [124]. However, clinical studies are still unavailable. Meanwhile, blocking excessive amounts of extracellular HMGB1, particularly the disulfide isoform, is an encouraging future clinical opportunity to ameliorate systemic inflammatory diseases. Therapeutic interventions to regulate intracellular HMGB1 biology must still await a deeper understanding of intracellular HMGB1 functions, and future research is warranted to evaluate functional bioactivity of HMGB1 antagonists. Forthcoming clinical studies will require the development of antibody-based assays to quantify HMGB1 redox isoforms, which are presently assessed by mass spectrometry methods [124].

Experimental post-sepsis studies demonstrated that RAGE mediates sepsis-triggered brain amyloid-β peptide accumulation and tau phosphorylation combined with cognitive impairment. These deleterious events are substantially prevented by repeated intracerebral injections of anti-RAGE antibodies in the hippocampus 2–3 weeks after the onset of sepsis, when the rats have clinically recovered from the acute-stage disease [197].

Apart from targeting alarmins, the downstream signaling molecules, activated upon release of alarmins, may be considered therapeutic targets. In particular, MAPKs represent promising targets, which are potentially useful in discovering new drugs with neuroprotective and anti-inflammatory properties. Among the MAPKs, JNK draws special attention considering that new specific JNK inhibitors have demonstrated neuroprotective potential in experimental research [44,45,47,80]. Since JNK3 is expressed in the brain and the heart, the development of selective inhibitors for this isoform may be promising, and therapeutic efficacy of new compounds should be studied in appropriate models of neuroinflammation.

## 16. Conclusions

**Figure 1:**
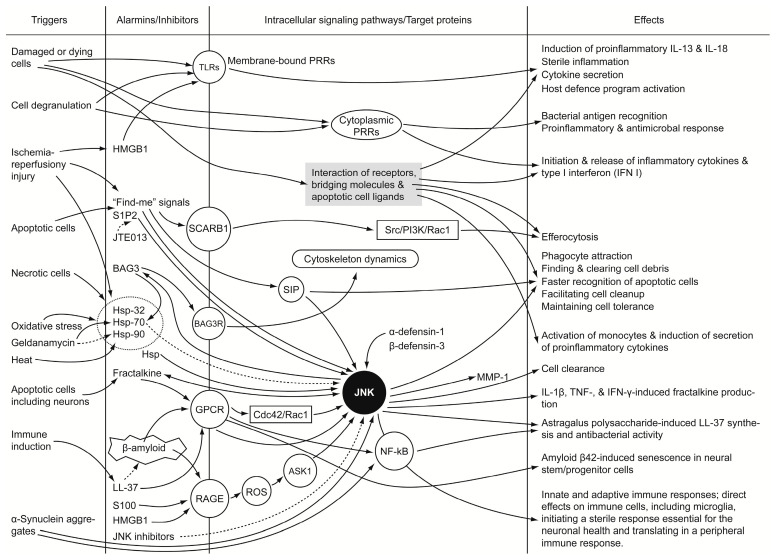
Schematic representation of alarmin signaling and interplay between the alarmin and JNK-signaling pathways. Solid lines indicate activation/induction processes; dashed lines indicate downregulation mechanisms.

While foreign pathogens and their products have long been known to activate the innate immune system, the recent recognition of a group of endogenous molecules that serve a similar function has provided a framework for understanding the overlap between the inflammatory responses activated by pathogens and injury. The endogenous alarmins are normal cell constituents that can be released into the extracellular milieu during states of cellular stress or damage and subsequently activate the immune system. Although alarmins contribute to the host’s defense, they promote pathological inflammatory responses. Alarmins are also known as DAMP, which are the tissue- and injury-specific molecular signatures serving as the markers of neurovascular inflammation and the targets for new promising anti-alarmin drugs. Studying alarmins and associated JNK signaling is promising regarding finding new therapeutic targets and downstream molecular markers of stressful events. The alarmins and signaling pathways more frequently associated with the neurodegenerative diseases comprise β-amyloid [26,158,159], α-synuclein [190,191], defensins [175], exogenous S100B [149,150], HMGB1 [130,131], Hsp [103], and IL-33 [155]. The origins, receptors and potential biological effects of alarmins in neuroinflammation are summarized in Table 1. β-Amyloid seems specific for the nervous tissue [26,158,159]. Among JNK isoforms, JNK3 is predominantly present in the brain, heart, and testicles [36]. New specific inhibitors of JNK warrant studies focusing on their effects on the processes induced by release of alarmins from injured cells. There are several factors hampering advancements of research in this field. First, there is no straightforward classification of alarmins available, whereas it is essential to classify the alarmins considering their diverse nature. Secondly, alarmins trigger the activation of many molecular cascades, which in parallel to JNK signaling involve other MAPKs and alternative signaling pathways (Figure 1). A coherent and holistic view of these processes is complex and requires a balanced and sophisticated research approach. Moreover, the mechanisms of how the cells are sensing injury of their microenvironment are also poorly understood. These processes may involve phenomena beyond the release of alarmins. Some of the mechanisms are specific, as certain alarmins have the unique receptors. Other mechanisms may be non-specific, which requires studying the detailed changes in physical-and-chemical homeostasis. Further studies on the role of alarmins in neuroinflammation and neurodegenerative diseases are warranted with a focus on downstream signaling pathways. It is also essential to elucidate, in detail, the timing and targets of alarmins in the activation of many molecular cascades in parallel to JNK.

## Figures and Tables

**Table 1:** The origins, receptors and potential biological effects of alarmins in neuroinflammation

Alarmin	Origin	Receptor	Potential Biological Effects	Ref.
HMGB1	Tissue damage	TLR4, RAGE	ROS-mediated JNK activation; NADPH-dependent ROS generation; oxidative stress signaling; autophagy; apoptosis; metabolic impairment	[119,120,121,122,123,124,125]
BAG3	Stressful stimuli	Hsc70/Hsp70 ATPase domain	Chaperone-assisted selective autophagy; hsp70-dependent and independent functions; maintaining the intracellular levels of anti-apoptotic factors and other molecules; protein quality control; cytoskeleton dynamics; structural and functional roles in myocytes	[139,140,141]
S100	Damaged cells	RAGE	ROS-mediated JNK activation; NADPH-dependent ROS generation	[125]
β-amyloid	AD pathogenesis	RAGE	ROS-mediated JNK activation; NADPH-dependent ROS generation	[125]
S1P	Activated platelets in the vasculature	Phagocytosis receptors, including MerTK and MFG-E8 on macrophages	Efficient phagocytosis; recruitment and priming of macrophages;	[75,76]
Spz5	Cell damage or necrotic death	Toll-1 receptor	Prepares, or primes, glia for phagocytosis in the CNS; activates M1-relevant ERK1/2 and JNK in post-ischemic brain	[72,77]
Fractalkine	Apoptotic neurons	CX3CR1	Activation of the proinflammatory pathway mediated by NF-κB as an early response in microglial cells	[78,79]
Hsp	Stressful conditions	TLR family	Inhibition of both aminoglycoside- and cisplatin-induced hair cell death in whole-organ cultures of utricles from adult mice	[112,113]
Hsp32	Trauma; hemorrhage; H_2_S preconditioning		Neuroprotection; mediation of the protective effect of celastrol; inhibition of pro-apoptotic JNK activation and hair cell death	[112]
Hsp70	Necrotic cells; paraquat-induced oxidative stress; caspase-3-mediated dopaminergic neuronal cell death	c-Type lectin receptors (CLR) and scavenger receptors (SR)	Reduction of paraquat-induced oxidative stress, JNK- and caspase-3-mediated dopaminergic neuronal cell death; decrease in the activated forms of JNK and p38 in the hippocampus of a rat model of fear memory consolidation	[107]
HSP/c70	Damaged astrocytes	TLR4	Activation of JNK in macrophage RAW264.7 cells	[106]
Hsp90	Stressful conditions	Glucocorticoid receptor	Neuroprotection	[114]
α-Synuclein	PD pathogenesis		Innate and adaptive immune responses; direct effects on immune cells, including microglia, initiating a sterile response essential for the neuronal health and translating in a peripheral immune response	[189,190]

## Abbreviations

AD	Alzheimer’s disease;
ADP	adenosine diphosphate;
ASK1	apoptosis-signal-regulated kinase 1;
BAG	Bcl-2-associated athanogene;
BAG3	BAG family molecular chaperone regulator 3;
BEAS	pulmonary epithelial cell line
CHDPs	cationic host defense peptides
CNS	central nervous system
DAMP	damage-associated molecular patterns
DNA	deoxyribonucleic acid
ERK	extracellular signal-regulated kinase
FPR2	N-formyl peptide receptor 2
GPCR	G-protein-coupled receptors
HMGB1	high mobility group box 1
Hsp	heat shock proteins
HSPA1L	heat shock protein family A member 1 like
IFN	interferon
IL	interleukin
IRS-1	insulin receptor substrate 1
JNK	c-Jun N-terminal kinase
LC3-II	light chain 3
LL-37	cathelicidin
LPS	lipopolysaccharide
MAPK	mitogen-activated protein kinases
MerTK	Mer receptor tyrosine kinase
MFG-E8	milk fat globule epidermal growth factor VIII
MMP	matrix metalloproteinase
MRP14	myeloid-related protein 14
mtDNA	mitochondrial deoxyribonucleic acid
NADPH	nicotinamide adenine dinucleotide phosphate
NF-κB	nuclear factor κ light chain enhancer of activated B cells
NLRP3	nucleotide-binding domain leucine-rich repeat (NLR) and pyrin domain containing receptor 3
NO	nitric oxide
PAMP	pathogen-associated molecular patterns
PARP-1	poly(ADP-ribose) polymerase-1
PD	Parkinson’s disease
PI3K	phosphoinositide 3-kinase
Rac1	Ras-related C3 botulinum toxin substrate 1
RAGE	receptor for advanced glycation end products
RNA	ribonucleic acid
ROS	reactive oxygen species
S100B	S100 calcium-binding protein B
SBT2171	exopolysaccharides produced by Lactobacillus helveticus
SCARB1	scavenger receptor class B type I
SDF-1	stromal cell-derived factor 1
SIP	sphingosine 1-phosphate
Spz5	protein spätzle
Src	proto-oncogene tyrosine-protein kinase
TLR	Toll-like receptor
TNF	tumor necrosis factor.
